# Preservation characteristics and bacterial communities of crimped ensiled barley grains modulated by moisture content and additive application

**DOI:** 10.3389/fmicb.2022.1092062

**Published:** 2022-12-21

**Authors:** Marcia Franco, Ilma Tapio, Marketta Rinne

**Affiliations:** Natural Resources Institute Finland (Luke), Jokioinen, Finland

**Keywords:** aerobic stability, animal feedstock, cereal grains, ensiling, feed preservation, *Hordeum vulgare*, microbiome, silage additive

## Abstract

Information about the relationships between preservation characteristics and main bacterial communities of fermented feeds can guide decision making during feed preservation and silage additive development. The objective was to evaluate fermentation quality, aerobic stability, microbial quality and bacterial profile of crimped barley grains ensiled under three moisture contents (MC): 228 (low MC), 287 (medium MC) and 345 (high MC) g/kg fresh matter; and using four additive treatments: 1. Control (CONT), 2. Formic and propionic acid-based additive (FPA), 3. Inoculation with homofermentative and heterofermentative strains of lactic acid bacteria (LAB), and 4. Salt-based additive (SALT). There was a quadratic effect (*p* < 0.05) of incremental MC on pH where greater decline happened from low (5.81) to medium (4.83) MC than from medium to high (4.28) MC, while lactic acid concentration and aerobic stability increased in a linear manner (*p* < 0.05). Ammonia-N and acetic acid concentrations increased quadratically (p < 0.05) with increasing levels of MC. The effects of additives depended on MC so that improvements in preservation characteristics in response to LAB and SALT were observed at medium and high MC, while FPA was effective at all MC levels. A minor shift was observed in bacterial ecology from raw material towards low MC samples, with *Erwiniaceae* sp., Enterobacterales spp. and *Pseudomonas* dominating the fermentation. A major change occurred in medium and high MC materials, where *Fructilactobacillus* dominated the fermentation in CONT, FPA and SALT silages. LAB-treated silages at medium and high MC resulted in a distinguished pattern with dominance of *Lentilactobacillus* followed by *Lactiplantibacillus*. Most abundant communities in the samples, such as *Fructilactobacillus*, *Erwiniaceae* sp., *Enterobacterales* spp. and *Pseudomonas*, were correlated with several fermentation characteristics. Our results showed that crimped barley grains could be successfully ensiled under various MC and additive treatments. Low MC feeds had higher risk to be aerobically unstable while high MC resulted in more extensive fermentation, with potentially poor fermentation quality. The suitable additive depends on the raw material characteristics as LAB and SALT require relatively high MC to be effective, while FPA showed consistent improvements over all MC levels used in the current study. Awareness of the MC of grain prior to ensiling allows to identify the risks to preservation quality and provides information for choosing an effective additive.

## Introduction

Cereal grains harvested under humid Northern European conditions are commonly artificially dried to secure a proper preservation during storage. However, it adds extra costs and energy requirement to the feed production system. Crimping and ensiling the moist grains offers farmers an additional advantage of harvesting cereals with less dependence on weather conditions, and at wider range of ripening stage than when conventionally harvested for drying. This may be increasingly valuable under the changing climate with more unpredictable weather condition during harvesting. Crimping has resulted in equal milk production as using dried grains in dairy cow feeding (Rinne et al., 2022). The growth performance of growing bulls was even increased, when high moisture instead of dry barley was used (Huuskonen et al., 2020). Hydrolysis of fibre during the fermentation may increase the grain digestibility in monogastric farm animals (Perttilä et al., 2001) and anti-nutritional compounds of grain legume seeds may be degraded during the in-silo fermentation (Rinne et al., 2020) providing additional benefits to this type of grain preservation.

The preservation of crimped ensiled grain is based on the same mechanism as with grass silage, i.e., lactic acid fermentation under anaerobic conditions (McDonald et al., 1991). The crimping process breaks and flattens the grain, which exposes the kernel’s endosperm to the microbes and contributes to greater compaction, creating a more anaerobic environment inside the silo. The moisture content (MC) of the raw material of crimped grain affects the microbial activity, and the drier the grain, the less fermentation occurs. Another important factor modulating the fermentation is the use of various additives such as biological products (lactic acid bacteria inoculants and enzymes) and chemicals (organic acids and salts) (McDonald et al., 1991; Muck et al., 2018).

Previously, knowledge of the microbiome of fermented feeds such as ensiled forages or crimped grains was obtained primarily through culture-dependent methods that underestimated the bacterial diversity. However, great efforts from the scientific community have been made in recent years to develop state-of-the-art next-generation sequencing technologies to characterize bacterial communities in silages (Romero et al., 2018). A detailed understanding of the bacterial community composition can provide insight into the relationship between fermentation quality parameters in silage and key bacterial communities (McAllister et al., 2018; Bai et al., 2022), which can guide decision making during silage production and development of novel more efficient silage additives. Therefore, several attempts to profile the bacterial communities have been performed focusing on lucerne silage (McGarvey et al., 2013; Guo et al., 2018; Wang et al., 2020; Li et al., 2022), timothy grass silage (Franco et al., 2022), sugarcane silage (Zheng et al., 2022), intercropped maize and soybean silage (Zeng et al., 2020), whole-plant maize silage (Gharechahi et al., 2017; Xu et al., 2021a), and barley whole crop silage (Liu et al., 2019). According to Franco et al. (2022), specific bacterial communities may modulate the nutritional composition, hygienic quality and the overall ensiling process of the feed as well as influence the silage losses.

The bacterial communities that are present in crimped barley grain raw material and in final feeds after fermentation are not yet characterized in depth but understanding them is of fundamental importance to explain the characteristics of the fermentation process, cause-and-effect relationships between bacterial communities and fermentation parameters, and consequently strategies to improve feed production systems. To our knowledge, no information can be found in literature about the effect of MC and silage additives on the links between fermentation quality and bacterial ecology of crimped barley grain. Therefore, the objective of the current study was to evaluate the bacterial profile, fermentation quality, aerobic stability and microbial quality of crimped barley grain ensiled under different management conditions, such as MC levels and application of different types of additives.

## Materials and methods

### Raw material for silage making

This experiment was conducted at the experimental facilities of the Natural Resources Institute Finland (Luke) in Jokioinen, Finland (60°48’N, 23°29′E). Barley (*Hordeum vulgare*) grain was combine harvested at Luke Jokioinen on 28th of August 2019. A representative raw material sample was immediately taken for the MC analysis in order to make a decision regarding the manipulation of the different MC levels. The MC of grains immediately after harvesting was 199 g/kg. Barley grain was crimped without additives using a farm scale crimper (MD 700 HD, Murska Ltd., Ylivieska, Finland). After crimping, a representative raw material sample was immediately taken for the analyses of chemical composition, microbial quality (enterobacteria, moulds and yeasts) and bacterial communities.

### Treatment and experimental procedures

The experiment was conducted according to a 3 × 4 factorial design, with three MC and four additive treatments. The crimped barley was divided into three batches and tap water was added at three levels to achieve the following MC: 228 (low MC), 287 (medium MC) and 345 (high MC) g/kg fresh matter.

The following silage additive treatments were used for each MC following the commercially recommended levels of application:

1. Control (CONT), distilled water
2. Formic and propionic acid-based additive (FPA) at 5 l/t of fresh matter (formic acid, propionic acid, sodium formate, and potassium sorbate; AIV Ässä Na, Eastman, Oulu, Finland)
3. Inoculation with homofermentative and heterofermentative strains of lactic acid bacteria (LAB) at 2.0 × 10^5^ cfu/g of fresh matter (*Lactobacillus plantarum* DSM 3676 and DSM 3677, and *Lactobacillus buchneri* DSM 13573; Kofasil Duo, Addcon, Bitterfeld-Wolfen, Germany)
4. Salt-based additive (SALT) at 4 l/t of fresh matter (sodium nitrite, sodium benzoate and potassium sorbate; Safesil Challenge, Salinity AB, Gothenburg, Sweden).

The additives were diluted in distilled water to have an even application (25 ml/3 kg crimped barley grain) and the total amount of liquid was the same for every treatment. The liquids were sprayed onto the crimped grain uniformly and mixed thoroughly by hand. For the control treatment, same amount of distilled water was applied. Each treatment was ensiled in triplicate and preserved in glass jars of 1.5 litre volume. To have enough material, two jars as sub-replicates were prepared per replicate. The glass jars were filled manually and pressed as full as possible to minimize the volume of air in the headspace resulting in densities of 545, 540 and 532 kg dry matter (DM)/m^3^ for low, medium and high MC, respectively. The top of the jar was covered with plastic film, closed airtight, weighed and stored at room temperature (20°C) in the dark for 81 days.

Jars were weighed before opening. The two sub-replicates were combined into one sample and mixed thoroughly. After that samples were taken for chemical analyses, microbial quality (enterobacteria, moulds and yeasts), bacterial communities and aerobic stability.

### Chemical composition, fermentation quality, microbial quality, and aerobic stability analyses

Chemical analyses were carried out at the Luke Laboratory in Jokioinen. The laboratory has a quality system which follows the SFS-EN ISO/IEC 17025:2005 standards and is accredited by FINAS (the Finnish Accreditation Service) with number T024. Samples for chemical composition and fermentation quality were stored in -20°C prior to analysis according to standard laboratory methods. The DM concentration was determined by drying at 105°C for 16 h and in case of fermented feeds, corrected for volatile losses (Huida et al., 1986). The MC was calculated as 1,000 – DM (g/kg). Ash (method 942.05) and crude protein (CP; Dumas method; method 968.06 using Leco FP 428 nitrogen analyser - Leco Corp., St Joseph, MI, United States and the correction factor 6.25 × N) were determined according to AOAC (1990). Concentration of ash-free neutral detergent fibre on organic matter basis (aNDFom) was determined in an ANKOM 220 Fiber Analyzer (ANKOM Technology, Macedon, NY, United States) according to Van Soest et al. (1991) using sodium-sulphite and α-amylase. Starch was determined according to Salo and Salmi (1968). Volatile fatty acids were determined according to Huhtanen et al. (1998) and the concentration of propionic acid was corrected for the amount added in the FPA using an 80% recovery rate. Lactic acid was determined according to Haacker et al. (1983), water soluble carbohydrates (WSC) according to Somogyi (1945) and ammonia-N according to McCullough (1967). The N content of the raw material before ensiling was used to express ammonia-N proportions in total N after fermentation. Ethanol was measured using an enzymatic kit (cat no.981680, KONE Instruments Corporation, Espoo, Finland) and the selective clinical chemistry analyser Pro 981,489 (KONE Instruments Corporation, Espoo, Finland) according to application instructions given by KONE. Buffering capacity of the raw material before ensiling was analysed according to Weissbach et al. (1974).

Samples for microbial quality were immediately analysed. The samples were mixed and 25 g was weighed in stomacher bags and mixed with 225 ml of ¼-strength Ringer solution (Merck 1.15525.0001, Merck KGaA, Darmstadt, Germany). The samples were homogenized with stomacher (Stomacher® 400 Circulator, Seward Ltd., Worthing, United Kingdom) for 2 min at 230 rpm. Serial decimal dilutions were prepared by mixing 1 ml of sample with 9 ml of Ringer solution. Yeasts and moulds were determined on Dichloran Rose Bengal Chloramphenicol Agar medium (LAB217, Lab M Ltd., Lancashire, United Kingdom) which was supplemented with 50 μg/ml of oxytetracycline hydrochloride (AppliChem BioChemica A5257, Darmstadt, Germany). The Petri dishes were incubated at 25°C. The colonies were counted after 3 and 5 d. Enterobacteria were determined on Violet Red Bile Glucose Agar medium (LAB088, Lab M Ltd., Lancashire, United Kingdom). The petri dishes were incubated at 37°C and the colonies were counted after 24 h.

Aerobic stability testing was carried out immediately after silo opening with approximately 700 g of silage samples in polystyrene boxes that allow air ingress. Thermocouple wires were inserted in the middle of the sample in the polystyrene boxes, which were connected to a data logger and temperature was automatically recorded at 10-min intervals. Aerobic stability was defined as the time taken to increase the temperature of the sample 2°C above the ambient temperature.

Silos were weighed immediately after filling and before opening for calculation of ensiling losses according to Knicky and Spörndly (2015) by assuming the weight loss to be CO_2_ leaving the silo during fermentation. It was supposed that for every mole of CO_2_ generated and released into the environment, 1 mol of H_2_O was produced. This means that for every gram of weight reduction due to CO_2_ loss, 0.44 g of DM in the silo was converted into water, which represents loss even though it remains inside the silo. Thus, DM loss was considered as the reduction in weight of the silo multiplied by a factor of 1.44, expressed in g/kg initial DM.

### DNA extraction, sequencing, and analysis of bacterial diversity

Samples for DNA extraction and sequencing were kept in -80°C prior to analyses. The DNA extraction was performed from 0.2 g of freeze dried and ground raw material and fermented grain samples following the protocol by Yu and Morrison (2004). Bacterial community composition was determined using universal primers 515F and 806R (Caporaso et al., 2011) for 16S rRNA gene V4 region amplicon sequencing. Sequencing library was prepared and sequenced in Finnish Functional Genomics Centre (Turku, Finland) on Illumina MiSeq platform by using 2 × 250 bp chemistry. Demultiplexing of sequences, adapter removal and sorting sequences by barcode were performed by the sequencing data provider. Sequencing data was further processed using QIIME 2 v 2022.8 (Bolyen et al., 2019). Briefly, quality control, filtering of chimeric reads, and clustering of bacterial sequences into amplicon sequence variants (ASV) were performed using DADA2 (Callahan et al., 2016). ASVs with the total abundance of less than 10 were removed. Bacterial ASV taxonomy was assigned using the Silva 138.1 database (Quast et al., 2012) by utilizing 515f-806r-uniform-classifier.qza (Bokulich et al., 2018) downloaded from https://zenodo.org/record/6395539. ASVs affiliated with mitochondria, chloroplast and Cyanobacteria were removed before further analysis.

### Data processing and statistical analyses

Data was analysed using a MIXED procedure of SAS (SAS Inc. 2002–2012, Release 9.4; SAS Inst. Inc., Cary, NC, United States) with MC, additive and their interaction as fixed effects, while replicate was used as a random effect. The Univariate procedure was used to test the normal distribution of data through Shapiro–Wilk test. Least squares means and standard errors of the means were reported per treatment and differences among treatment means were declared significant at 5% of probability. The linear and quadratic effects of MC were conducted using contrasts. In addition, pairwise comparisons among all treatment means were performed using Tukey’s test at a probability level of *p* < 0.05.

Silage bacterial community alpha diversity was evaluated using Shannon and Simpson diversity indexes as well as observed number of ASVs. To evaluate treatment effect on the changes in silage bacterial community structure, between sample diversity was calculated as Bray-Curtis dissimilarities following Hellinger transformation and visualized using principal co-ordinate analysis (PCoA) as implemented in *MicrobiotaProcess* R package (Xu and Yu, 2021). The significance of groups was evaluated by distance-based permutational multivariate analysis of variance (adonis) and defined at *p* < 0.05 level after 999 permutations, as implemented in *vegan* R package (Oksanen et al., 2019). To determine which bacterial taxa were affected by the crimped barley grain silage preservation and management methods, a linear discriminant analysis was performed as implemented in *MicrobiotaProcess*. Significance was defined at *p* < 0.05 with false discovery rate correction (*q* < 0.05).

To explore the magnitude of associations between bacterial communities and silage fermentation characteristics, the variables were ordered based on an analysis of a Spearman correlation plot (CORR procedure of SAS) and a heat map originated from two-dimensional display was created to characterize the effects of bacteria species on fermentation characteristics. Correlation data was filtered so that all genera below 0.1% in up to 50% of the samples were left out. This filtering reduced the number of genera from 60 to 24.

## Results and discussion

### Raw material characteristics

The chemical composition and microbial quality of the crimped barley grain before MC manipulation is shown in Table 1. The MC of the cereal grains varies greatly depending on the stage of ripening and the prevailing weather conditions. Under Finnish climatic conditions, the grains need to be artificially dried to achieve the MC of 140 g/kg used as a default for dry grains, while the practical recommendation for effective grain preservation by fermentation is above 300 g/kg. Olstorpe et al. (2010) reported MC of seven crimped grain farm samples in Sweden to range from 150 to 300 g/kg, but noted that the weather conditions had been exceptionally dry and MC of most samples below the optimum in that data set. As an example, the MC of faba bean seeds under moist weather conditions was as high as 443 g/kg when combine harvested in October under Finnish conditions (Rinne et al., 2020). By adding water into a single batch of grains, we could mimic the effects of moisture content on the microbial activity, when all other factors were kept constant, but in real life, the decreasing moisture content and increasing ripening as well as changes in epiphytic microbiota happen concomitantly, although in a way that can be sometimes arbitrarily modified by the environmental conditions. The CP, starch, ash and aNDFom concentrations of the barley grains were typical (Luke, 2022). According to Kristensen et al. (2010), yeast and mould contamination must be below 10^6^ for the raw material to be considered of adequate microbiological quality, which in the case of this experiment was achieved. Additionally, according to Wilkinson and Davies (2013), the low counts of yeasts and moulds of the raw material during harvest are key factors to increase the aerobic stability of silage when it is exposed to aerobic conditions during the feedout period.

**Table 1:** Composition and microbial quality of crimped barley grain before ensiling.

	Crimped barley
Moisture content, g/kg	199
Buffering capacity, g lactic acid/100 g DM	0.93
pH	6.04
In dry matter (DM), g/kg	
Ash	33
Crude protein	117
Starch	542
Neutral detergent fibre	215
Microbial quality, cfu/g	
Yeasts	1.8 × 10^6^
Moulds	1.9 × 10^5^
Enterobacteria	1.3 × 10^6^

cfu, colony-forming units.

### Preservation characteristics of the experimental silages

Higher moisture content of the feed promotes microbial activity (Kung, 2010), which resulted in a linearly (*p* < 0.05) increasing lactic acid concentration of the current materials with increasing MC. The subsequent effect on pH was quadratic (*p* < 0.05) as a greater decline happened from low (5.81) to medium (4.83) MC than from medium to high (4.28) MC (Table 2). Also acetic and butyric acids as well as ethanol concentrations increased with increasing MC, and the amounts of total fermentation acids increased in a quadratic manner (*p* < 0.05) with greater increase from medium to high MC. Ammonia-N concentration increased quadratically (*p* < 0.05) with increasing levels of MC, with greater intensity from medium MC to high MC than from low to medium MC. Ammonia-N is generated during proteolysis that occurs through the action of plant and microbial proteases, inevitably linked with silage fermentation (Hassanat et al., 2007), and the lower the better. These responses to MC are typical for crimped grains (Olstorpe et al., 2010; Rinne et al., 2020) as well as for grass silages (Guo et al., 2020).

**Table 2:** Chemical composition, fermentation quality, ensiling losses and microbial quality of crimped barley grain ensiled under different moisture content (MC) levels and treated with additives.

Moisture content^1^	Low MC				Medium MC					High MC				SEM^3^	*p*-value^4^			
Additive^2^	CONT	FPA	LAB	SALT	CONT	FPA	LAB	SALT		CONT	FPA	LAB	SALT		MClin	MCquad	Add	MC × Add
Moisture content, g/kg	238^f^	235^f^	238^f^	238^f^	298^d^	291^e^	292^e^	295^de^		357^a^	350^bc^	347^c^	354^ab^	0.9	<0.001	0.116	<0.001	0.002
pH	6.16^a^	4.88^bc^	5.97^a^	6.24^a^	5.25^b^	4.54^cd^	4.25^de^	5.29^b^		4.56^cd^	4.19^de^	4.03^e^	4.34^d^e	0.084	<0.001	<0.001	<0.001	<0.001
Ammonia-N, g/kg N	6.8^g^	5.3^g^	6.4^g^	5.3^g^	16.4^cd^	9.3^f^	17.5^c^	14.6^de^		38.3^a^	14.3^e^	29.2^b^	28.1^b^	0.35	<0.001	<0.001	<0.001	<0.001
Ethanol, g/kg dry matter (DM)	6.5^b^	0.1^f^	6.5^b^	3.8^d^	9.5^a^	0.4^f^	5.8^bc^	4.9^c^		9.6^a^	2.0^e^	5.6^bc^	6.2^b^	0.19	<0.001	0.304	<0.001	<0.001
Acids, g/kg DM																		
Lactic (LA)	0.6^e^	0^e^	1.3^de^	0.3^e^	6.2^c^	1.1^de^	19.1^b^	5.1^cd^		16.8^b^	6.4^c^	27.3^a^	17.5^b^	0.86	<0.001	0.096	<0.001	<0.001
Acetic (AA)	0.9^e^	0.8^e^	1.1^de^	1.0^e^	2.1^d^	1.4^de^	4.4^bc^	2.1^d^		4.2^c^	3.5^c^	5.3^ab^	5.6^a^	0.20	<0.001	0.025	<0.001	<0.001
Propionic^5^	0.12^a^	0.07^a^	0.11^a^	0.12^a^	0.13^a^	0^b^	0.12^a^	0.11^a^		0.12^a^	0^b^	0.12^a^	0.12^a^	0.012	0.052	0.199	<0.001	0.031
Butyric	0.02^b^	0^b^	0.01^b^	0^b^	0.03^b^	0^b^	0.01^b^	0^b^		0.44^a^	0.01^b^	0.07^b^	0.01^b^	0.026	<0.001	<0.001	<0.001	<0.001
Total volatile fatty acids	1.1^f^	0.9^f^	1.3^f^	1.1^f^	2.3^e^	1.4^ef^	4.5^c^	2.2^e^		4.8^bc^	3.5^d^	5.6^ab^	5.8^a^	0.18	<0.001	0.002	<0.001	<0.001
Total fermentation acids^6^	1.6^e^	0.9^e^	2.5^de^	1.3^e^	8.4^c^	2.5^de^	23.6^b^	7.3^cd^		21.6^b^	10.0^c^	32.9^a^	23.3^b^	1.04	<0.001	0.049	<0.001	<0.001
Total fermentation products^7^	8.1^def^	1.0^g^	9.0^de^	5.1^efg^	17.9^c^	2.9^fg^	29.4^b^	12.2^cd^		31.2^b^	12.0^cd^	38.5^a^	29.5^b^	1.17	<0.001	0.108	<0.001	<0.001
LA/AA ratio	0.62^ghi^	0.02^i^	1.16^fg^	0.27^hi^	2.83^cd^	0.72^gh^	4.36^b^	2.39^de^		4.04^b^	1.83^ef^	5.15^a^	3.11^c^	0.134	<0.001	<0.001	<0.001	<0.001
Aerobic stability^8^	45^c^	265^ab^	43^c^	74^c^	58^c^	269^ab^	198^abc^	149^bc^		206^abc^	360^a^	151^bc^	349^a^	34.2	<0.001	0.394	<0.001	0.019
Ensiling losses, g/kg of initial DM	7.0^bc^	0.7^e^	7.0^bc^	4.8^cd^	10.0^ab^	2.6^de^	7.7^bc^	5.7^cd^		12.3^a^	2.8^de^	7.4^bc^	6.9^bc^	0.63	<0.001	0.338	<0.001	0.035
Yeasts, cfu/g	3.8 × 10^2b^	1.0 × 10^6a^	2.8 × 10^2b^	2.8 × 10^3b^	2.3 × 10^2b^	3.7 × 10^3b^	2.0 × 10^2b^	1.1 × 10^4b^		5.0 × 10^2b^	4.0 × 10^2b^	1.0 × 10^2b^	2.4 × 10^4b^	1.5×10^5^	0.034	0.196	0.032	0.012
Moulds, cfu/g	1.7 × 10^6b^	1.0 × 10^2c^	3.6 × 10^6a^	5.6 × 10^4c^	1.0 × 10^6bc^	1.0 × 10^2c^	5.8 × 10^4c^	1.3 × 10^3c^		8.7 × 10^4c^	1.0 × 10^2c^	9.5 × 10^4c^	1.0 × 10^2c^	2.3×10^5^	<0.001	<0.001	<0.001	<0.001
Enterobacteria, cfu/g	2.7×10^4^	1.0×10^5^	5.5×10^4^	6.5×10^2^	6.6×10^1^	1.0×10^1^	1.0×10^1^	1.0×10^1^		1.0×10^1^	1.0×10^1^	5.0×10^1^	1.0×10^1^	3.2×10^4^	0.053	0.251	0.613	0.716

^1^Moisture contents, Low MC: 228 g/kg; Medium MC: 287 g/kg; High MC: 345 g/kg.

^2^Additives, CONT: control; FPA: formic and propionic acid-based additive; LAB: inoculation with homolactic and heterolactic acid bacteria strains; SALT: salt-based additive.

^3^SEM: Standard error of the mean.

^4^MClin: linear effect of moisture content; MCquad: quadratic effect of moisture content; Add: effect of silage additive; MC×Add: interaction between moisture content and additive.

^5^Propionic acid was corrected for its amount added via FPA additive.

^6^Total volatile fatty acids + lactic acid.

^7^Total fermentation acids + ethanol.

^8^Time taken (in hours) to increase the temperature of samples by 2°C above the ambient temperature (22°C).

cfu: colony-forming unit, estimates the number of viable bacteria or fungal cells in a sample.

Values with different superscript letter in a row were significantly different at 5% Tukey test. If there were no differences in Tukey test, letters were removed.

Using additives was effective in reducing pH (*p* < 0.05) of crimped ensiled grain and also in reducing (*p* < 0.05) the concentrations of ammonia-N and ethanol (Table 2). Although all additives were efficient in reducing the concentrations of ammonia-N and ethanol, FPA resulted in the most significant reductions in both parameters. All additives were effective in decreasing (*p* < 0.05) the butyric acid concentration of the samples at high MC when compared to CONT. The effects of additives depended on MC so that improvements in preservation characteristics in response to LAB and SALT were observed at medium and high MC, while FPA was effective at all MC levels. The efficacy of formic acid-based additives to restrict fermentation and improve the preservation quality has been observed in several earlier studies preserving high-moisture grains (Franco et al., 2019; Huuskonen et al., 2020; Rinne et al., 2020). The effects of additives on the extent of fermentation were rather typical for ensiled grains, in a way that LAB boosted the fermentation, while FPA restricted it, and SALT had a minor effect on it as also observed by Rinne et al. (2020) using simultaneously all these types of additives on crimped ensiled faba bean seeds.

Aerobic stability linearly improved (*p* < 0.05) being 107 h at low MC, 169 h at medium MC and 267 h on high MC, which can be explained by increased concentrations of fermentation end products with antimicrobial activities in the moister materials. According to McDonald et al. (1991), the absence of lactic acid in low MC raises the pH of the crimped grain to a level that allows the growth of opportunistic bacteria, and subsequently reduces the quality of the silage. In addition, the air ingress in the drier feed material may facilitate the growth of aerobic bacteria in-silo.

The average aerobic stabilities for the additives CONT, FPA, LAB, and SALT were 103, 298, 131 and 190 h, respectively. The production of lactic acid is largely responsible for the desired drop in pH, but this acid has poor anti-fungal activity, unlike acetic and propionic acids. For this reason, compared to the CONT, LAB increased lactic acid fermentation, but no improvement was observed in the aerobic stability (Table 2). When silage is exposed to air during the feeding phase, lactic acid begins to be oxidized by lactate assimilating yeasts (Pahlow et al., 2003). This results in an increase in pH providing improving conditions for various aerobic microbes. Therefore, silages with a high amount of lactic acid in relation to acetic acid are prone to a short aerobic stability when exposed to aerobic conditions. In our experiment, FPA was the only additive that was effective in improving aerobic stability also at the lowest MC level, probably through the antimicrobial effect of the propionic acid included in the additive.

As stated by Wilkinson and Davies (2013), the aerobic stability of feeds is the main factor to secure that the feed is properly preserved and harmless in terms of least possible presence of moulds before offering it to ruminants. Rinne et al. (2022) indicated that total mixed rations prepared with ensiled crimped barley grains silage could potentially decrease the aerobic stability when compared with the use of dry barley. This finding reinforces the need for proper preservation strategies to overcome future shortcomings during the feeding phase.

The weight losses during the fermentation phase linearly increased (*p* < 0.05) with increasing MC level of the crimped barley grain, which can be directly linked with the more intensive fermentation of them. Generally, the DM portion lost during fermentation is of high nutritional quality, as it is the most digestible part of the feed. The ensiling losses could however be controlled with the use of additives (*p* < 0.05) when compared to CONT. FPA was the most efficient in reducing losses, while LAB and SALT showed intermediate results. Consistent with the present experiment, Franco et al. (2019) found that the use of organic acids in the preservation of ensiled crimped wheat grains was efficient in reducing the extent of fermentation and subsequent ensiling losses. According to Rocha et al. (2014), an additional major problem to the ensiling losses is the need to discard feed and especially the potential mycotoxins production, which pose serious risks to animals, their production and potentially even humans.

Proper management factors at the time of silage making are more important and efficient in controlling the development of microorganisms than later during the preparation of the total mixed ration or at the feed bunk (Kung, 2010). Enterobacteria numbers were lower in all treatments and MC levels in relation to the raw material prior ensiling so that effective feed fermentation can serve as a means to control the microbial counts. Yeasts of FPA at low MC were similar to the raw material, but lower for all other treatments. The yeast count to avoid reductions in the aerobic stability of the feed has been established at 10^5^ cfu/g (Borreani and Tabacco, 2010). Considering this threshold, most treatments remained below this level, except for FPA at low MC. However, this did not negatively impact the aerobic stability of the FPA crimped barley grain silage at low MC probably due to the protective effect of propionic acid included in it. The mould counts were higher than in the raw material for the CONT and LAB treatments in the low MC and also CONT in the medium MC, while these counts were lower than the raw material for all other treatments.

### Bacterial communities in the experimental silages

After quality filtering and removal of chimeric reads there were 654,711 sequencing reads in total with on average 15,225 reads per sample. One sample treated with FPA at the low MC was excluded from alpha diversity analysis because it had only 1800 reads.

The alpha diversity estimates of the raw material and the ensiled samples are presented in Table 3. The grain raw material showed greater alpha diversity than most of the ensiled samples for all indexes evaluated, which is in line with Fu et al. (2022), who investigated bacterial ecology in ryegrass raw material and silages. Additionally, there was a decreasing linear effect in alpha diversity with increasing barley grain MC in all additive treatments except CONT. Clear differences were observed between additive treatments, as LAB showed the highest diversity of bacterial communities, followed by SALT and CONT, while FPA showed the lowest alpha diversity. Partially in agreement with our experiment, Liu et al. (2019) reported that the diversity of bacterial communities also decreased for both CONT and LAB inoculated barley whole-crop silages, but unlike our experiment, the decrease had greater magnitude in LAB-treated silages. FPA treated samples in the highest MC level presented the lowest diversity of bacterial communities for all indexes evaluated. High MC level reduced the richness of bacterial communities in all additive treatments except CONT, and the greatest decrease happened for SALT (*p* = 0.027) with concomitant increase of the MC. LAB treatment at the low MC tended to have richer bacterial community diversity as compared to medium or high MC levels (*p* = 0.061), while CONT and FPA were not affected (*p* = 0.240 and *p* = 0.098, respectively) by the MC level when investigating the Observed ASV estimate. At the medium MC a similar effect on bacterial richness measured by the Observed ASV of all additives were detected (*p* = 0.340), while low and high MC levels affected the additives in different ways (*p* = 0.046 and *p* = 0.020, respectively). For instance, SALT had numerically lower richness at high MC and higher at low MC, while CONT behaved in an opposite direction.

**Table 3:** Alpha diversity estimates of crimped barley grain ensiled under different moisture content (MC) levels and treated with additives.

Moisture content^1^	Raw material	Low MC				Medium MC				High MC				SEM^3^	*p*-value^4^			
Additive^2^		CONT	FPA	LAB	SALT	CONT	FPA	LAB	SALT	CONT	FPA	LAB	SALT		MClin	MCquad	Add	MC × Add
Observed ASV^5^	82.0	71.3^ab^	57.6^bc^	82.0^a^	82.7^a^	66.7^abc^	56.0^bc^	68.7^ab^	62.7^bc^	81.7^a^	48.3^c^	62.3^bc^	54.0^bc^	3.57	<0.001	0.085	<0.001	0.001
Shannon	3.85	3.71^ab^	3.57^abc^	3.87^a^	3.78^a^	3.37^abcd^	3.06^cde^	3.66^ab^	3.24^bcd^	3.40^abcd^	2.66^e^	3.53^abc^	2.89^de^	0.099	<0.001	0.147	<0.001	0.037
Simpson	0.959	0.953^ab^	0.953^abc^	0.960^ab^	0.953^abc^	0.942^abc^	0.926^bcd^	0.966^a^	0.935^abc^	0.941^abc^	0.897^d^	0.961^ab^	0.912^cd^	0.0070	<0.001	0.809	<0.001	0.021

^1^Moisture contents, Low MC: 228 g/kg; Medium MC: 287 g/kg; High MC: 345 g/kg.

^2^Additives, CONT: control; FPA: formic and propionic acid-based additive; LAB: inoculation with homolactic and heterolactic acid bacteria strains; SALT: salt-based additive.

^3^SEM: Standard error of the mean.

^4^MClin: linear effect of moisture content; MCquad: quadratic effect of moisture content; Add: effect of silage additive; MC × Add: interaction between moisture content and additive.

^5^Observed amplicon sequence variants.

Values with different superscript letter in a row are significantly different at 5% Tukey test.

**Figure 1:**
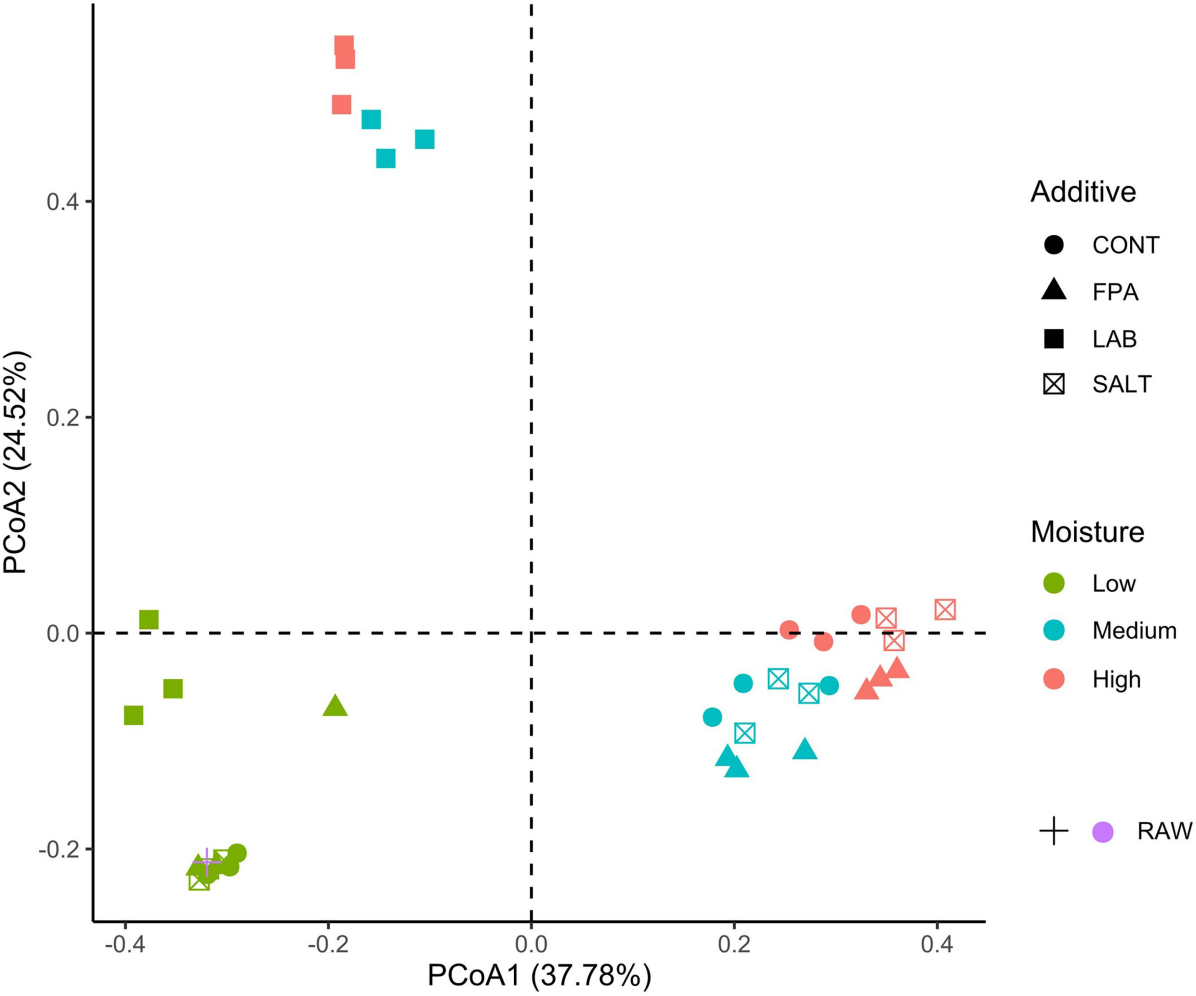
Principal coordinate analysis (PCoA) of the beta diversity analysis of crimped barley grain ensiled under different moisture content levels and treated with additives. CONT: control, FPA: formic and propionic acid-based additive, LAB: inoculation with homolactic and heterolactic acid bacteria strains, SALT: salt-based additive, Low: low moisture content, Medium: medium moisture content, High: high moisture content, RAW: raw material prior to ensiling.

The bacterial community structure in the crimped barley grain raw material prior to ensiling and in samples after preservation are shown in Figure 1. The MC level significantly affected the bacterial community structures of the crimped ensiled grains (adonis test *p* < 0.001; *R*^2^ = 0.78). The combined evaluation of both axes in the ordination plot discriminated samples produced under low MC separated from the other MC levels, but together with the raw material before ensiling. In other studies using grass silages (Franco et al., 2022; Fu et al., 2022), the raw material was identified separately from the samples after fermentation, forming a cluster of its own, which differs from our result, since here the raw material was grouped together with low MC silages. This indicates that the bacterial community structures of raw material and low MC silages were very similar, with a minor difference for LAB treated silages at low MC, which clustered a little apart, but still close to the raw material and other low MC silages. This can be explained by the limited microbial activity in low MC crimped grains. Interestingly, ensiled grain samples under medium and high MC levels were grouped together, but still divided in two points of the ordination plot. While most additives were grouped based on MC level, LAB samples remained separate from the rest in all MC groups, which suggests that the bacterial community structure of samples treated with this additive was different compared to the rest.

**Figure 2:**
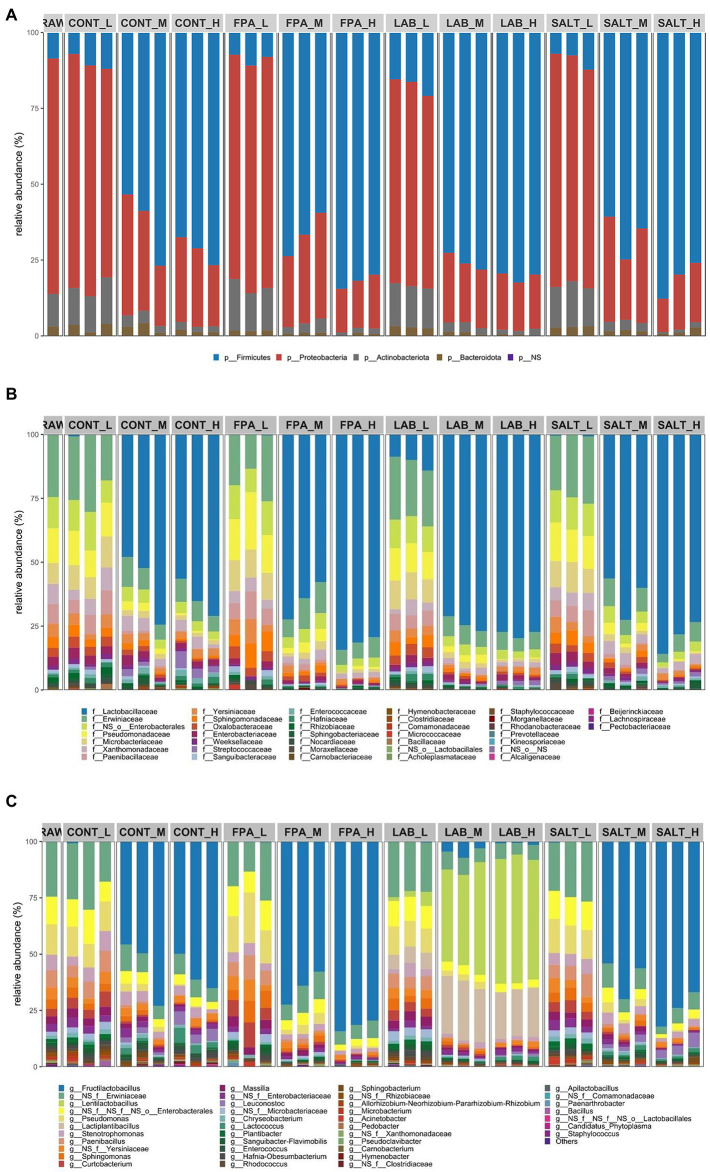
Relative abundance of bacterial communities at phylum **(A)**, family **(B)** and genus **(C)** taxonomic levels of crimped barley grain ensiled under different moisture content levels and treated with additives. CONT: control, FPA: formic and propionic acid-based additive, LAB: inoculation with homolactic and heterolactic acid bacteria strains, SALT: salt-based additive, L: low moisture content, M: medium moisture content, H: high moisture content, RAW: raw material prior to ensiling.

The relative abundance of bacterial communities in crimped ensiled grains was affected by the MC, but not necessarily by the additive treatments (Figure 2). Relative abundance of bacterial communities in raw material and preserved samples were dominated by Firmicutes, Proteobacteria, Actinobacteriota and Bacteroidota phyla (Figure 2A). Overall, at any ensiling management condition, Firmicutes and Proteobacteria were the most abundant phyla. The relative abundance of bacterial communities in the raw material was mostly dominated by Proteobacteria and equal portions of Firmicutes and Actinobacteriota, and remarkably this composition remained the same for all preserved samples produced with low MC, regardless of the additive. Apparently, this means that the composition of samples produced at low MC did not significantly alter their communities, thus remaining relatively similar to the raw material before ensiling. On the other hand, the samples produced with medium and high MC showed a pattern of relative abundance of bacterial communities different from the raw material and low MC, but even so additives did not exert significant effects, as these samples were mostly dominated by Firmicutes, followed by Actinobacteriota. Thus, the ensiling process caused a clear compositional change in the relative abundance of bacterial communities only for the samples produced under medium and high MC when compared to the epiphytic communities in the fresh raw material before ensiling. Environmental conditions developed during the ensiling of medium and high MC crimped barley grains contributed to the growth of the Firmicutes phylum. The transition between Firmicutes and Proteobacteria from raw material to the fermented feeds in this study seems to be quite consistent with several other studies (McGarvey et al., 2013; Keshri et al., 2018; Franco et al., 2022; Fu et al., 2022; Wang et al., 2022). The relative abundance of Actinobacteriota was significantly lower in samples produced under medium and high MC, than in samples produced with low MC. There were no significant differences regarding the application of different additives.

The crimped grain raw material showed a great variation in the relative abundance of families (Figure 2B), with *Erwiniaceae* dominating the composition, followed by Enterobacterales spp. and *Pseudomonadaceae*, and also lower abundance of *Microbacteriaceae*, *Xanthomonadaceae* and *Paenibacillaceae*. In the ensiled samples, relative abundance of families produced under low MC level resulted in similar proportions to the raw material, except for LAB treated samples, as these also presented a significant abundance of *Lactobacillaceae*. Although *Lactobacillaceae* was not found in the raw material, for crimped barley samples produced with medium and high MC grains, this community dominated the fermentation, regardless of the additive used. In these samples, the *Lactobacillaceae* community was followed in smaller proportions by *Erwiniaceae*, Enterobacterales spp. and *Pseudomonadaceae*. In concordance with the results of Von Gastrow et al. (2022), *Lactobacillaceae* was also not found in recently harvested wheat grain, but it was widely present in other stages of the production chain. The different additives had the same pattern regarding the relative abundance of communities within each MC level and no noticeable difference was observed between additives.

The *Fructilactobacillus* genus was not identified in the raw material (Figure 2C), nonetheless it mostly dominated the fermentation of grains produced with medium and high MC in CONT, FPA and SALT silages. Considering that *Fructilactobacillus* was formerly classified as *Lactobacillus* makes our results in line with Carvalho-Estrada et al. (2020), who found that *Lactobacillus* was the main bacterial genera in ensiled high-moisture maize grains. Fermentation of LAB-treated silages at the medium and high MC was dominated by *Lentilactobacillus* followed by *Lactiplantibacillus*, while for low MC, it resulted in more diversified populations with greater dominance of *Erwiniaceae* sp. Lactic acid bacteria genera were found in negligible amounts in the raw material, which suggests the need for inoculation or application of any other silage additive to boost or modulate the appropriate fermentation process. Low MC led to much more diverse populations than medium and high MC crimped barley samples. Raw material and low MC ensiled grains in all additives, in addition to *Erwiniaceae* sp. and Enterobacterales spp., also showed a significant amount of *Pseudomonas*. On the other hand, *Pseudomonas* was found in negligible quantity in samples produced with medium and high MC. Von Gastrow et al. (2022), similarly to our study, found that wheat grains shortly after harvest also had ample relative abundance of *Pseudomonas*, *Erwinia*, *Massilia*, *Paenibacillus* and *Sphingomonas*. Again, the different additives, except LAB at medium and high MC, had the same pattern regarding the relative abundance of genera communities within each MC level and no distinguished differences were observed between additives.

### Correlations between relative abundance of bacterial communities and silage fermentation characteristics

**Figure 3:**
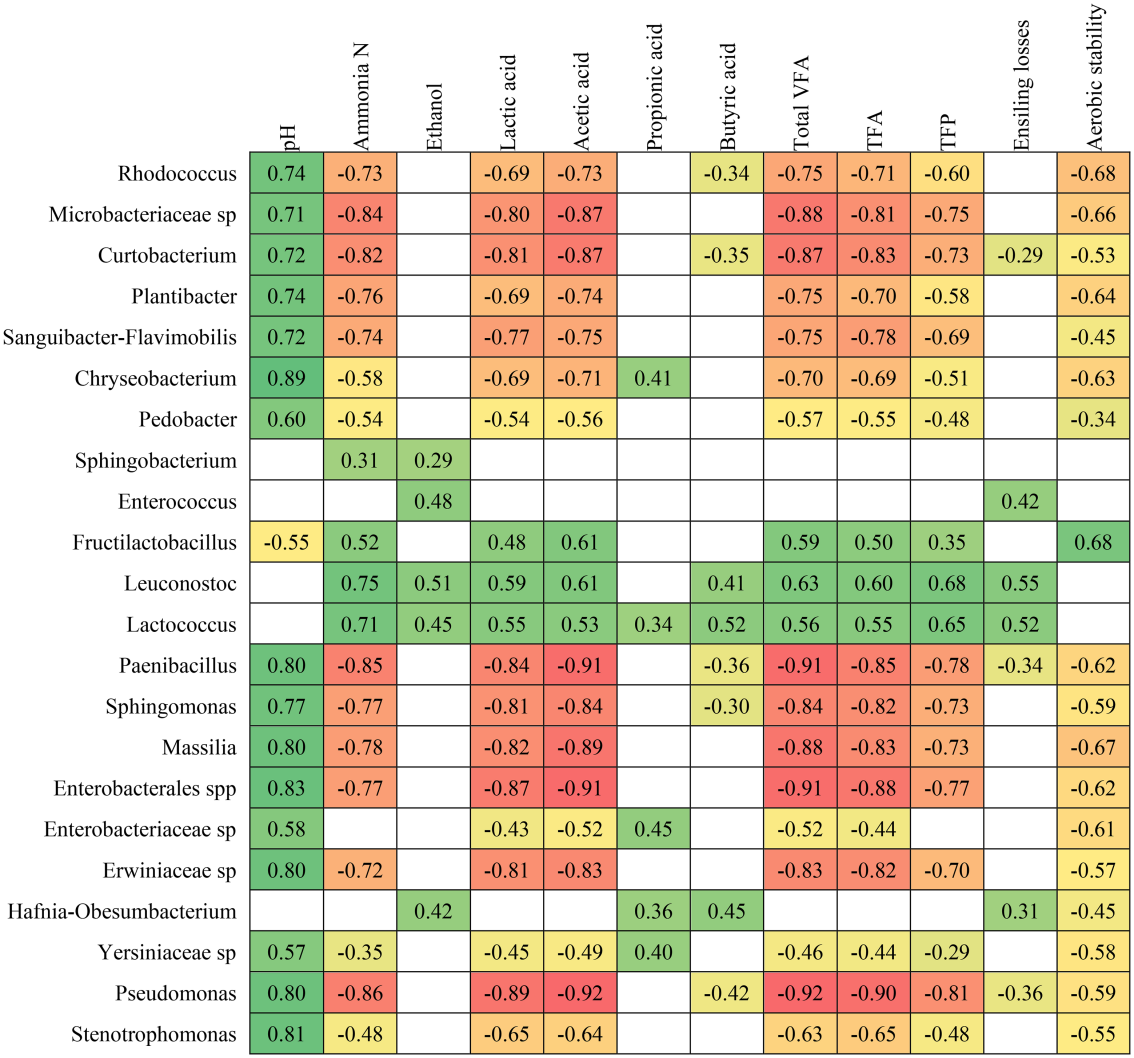
Spearman correlations between bacterial communities and preservation characteristics of crimped barley grain ensiled under different moisture content levels and treated with additives. VFA: volatile fatty acids, TFA: total fermentation acids, TFP: total fermentation products. From green (strong and positive correlation) to red colour (strong and negative correlation), while empty cells indicate a non-significant correlation.

The ensiling process is characterized by its complexity and interactions between parameters related to fermentation quality and bacterial communities. A Spearman’s correlation was performed to identify the relationships between the bacterial communities at the genus level and the parameters related to the silage fermentation characteristics (Figure 3). Although *Rhizobiaceae* sp. and *Allorhizobium-Neorhizobium-Pararhizobium-Rhizobium* were present at abundance above filtering threshold, they were not correlated with the silage fermentation characteristics, and therefore not included in Figure 3.

The pH of the ensiled grains showed a strong and positive correlation with most communities (Figure 3), such as *Rhodococcus*, *Microbacteriaceae* sp., *Curtobacterium*, *Plantibacter*, *Sanguibacter-Flavimobilis*, *Chryseobacterium*, *Pedobacter*, *Paenibacillus*, *Sphingomonas*, *Massilia*, *Enterobacterales* spp., *Enterobacteriaceae* sp., *Erwiniaceae* sp., *Yersiniaceae* sp., *Pseudomonas* and *Stenotrophomonas*. However, only one community showed a negative correlation with the pH, which was *Fructilactobacillus*. Franco et al. (2022) emphasized that the correlation between bacterial communities and fermentative characteristics of silage may imply that bacteria boost or restrict the amount of fermentation product, or vice versa. This can be beneficial or detrimental to the silage fermentation process considering the direction of effect on specific parameters as for instance, most of the communities positively correlated with pH were concomitantly negatively correlated with ammonia-N production. The first factor is detrimental since these communities being abundant in the silage might imply that the resulting silages have a higher pH. However, the second factor is positive, since these communities may be associated with low proteolysis of the crude protein and thus results in less formation of ammonia-N. The pattern that these communities exerted on ammonia-N was the same as on the production of lactic acid, acetic acid, total volatile fatty acids, total fermentation acids, total fermentation products and aerobic stability. It is possible to hypothesise that these communities, when present in large abundance in silages, may be associated with beneficial factors such as less production of ammonia-N, acetic acid and many unfavourable fermentation products. However, the drawback refers to the lower production of lactic acid that would be beneficial for obtaining low pH and shorter aerobic stability when the silage is air exposed. The longer the aerobic stability of a fermented feed, the better, as it means that the silage remains cool and microbiologically stable to be fed to animals. Nonetheless, most of the bacterial communities were negatively correlated with aerobic stability, which suggests shorter aerobic stability when these genera are abundant in crimped ensiled barley grains.

Among the above-mentioned genera, Ogunade et al. (2018) indicated that *Sphingomonas* could potentially be further studied at species level and their roles in the fermentation quality of silages. In their study, *Sphingomonas* acted in a beneficial way, as it was negatively correlated with silage pH and ammonia-N, possibly contributing to true protein preservation in lucerne silages. This argument partly agrees with our results, as in the current study, *Sphingomonas* also had a negative correlation with ammonia-N, but positive with pH. A similar positive correlation was found between Sphingomonas and pH by Franco et al. (2022).

*Sphingobacterium* was a genus positively correlated with only two fermentation quality parameters (Figure 3), ammonia-N and ethanol, as well as *Enterocuccus*, also positively correlated with ethanol and ensiling losses. These factors allow us to suppose that these communities in large presence in the silage are detrimental to the production system, as they imply greater proteolysis of the crude protein, greater formation of ethanol and greater losses during ensiling. Franco et al. (2022) studying bacterial ecology in timothy grass silages also found a positive correlation between the abundance of *Enterococcus* and ethanol concentration. Additionally, the genera *Leuconostoc* and *Lactococcus* were strongly and positively correlated with concentrations of ammonia-N, ethanol, lactic, acetic and butyric acids, total volatile fatty acids, total fermentation acids, total fermentation products and ensiling losses. Therefore, these communities might be harmful to all fermentation characteristics evaluated, since the less of most of them, the better, except for lactic acid.

The genus *Hafnia-Obesumbacterium* is clearly undesirable in the silage fermentation process (Figure 3), as it is correlated with greater production of ethanol, propionic and butyric acids, which are undesirable, as well as greater ensiling losses. In addition to all these negative features for the genus *Hafnia-Obesumbacterium*, it may also be associated with shorter periods of aerobic stability. Zhao et al. (2021) also identified that when *Hafnia-Obesumbacterium* was among the dominant genera in the fermentation process of lucerne silages with high MC, the silages tended to have a poor fermentative quality.

Butyric acid is an undesirable product during the fermentation process of silages (Vissers et al., 2007) and the communities that are negatively correlated with this product are beneficial. The beneficial communities that were negatively correlated to the production of butyric acid were *Rhodococcus*, *Curtobacterium*, *Paenibacillus*, *Sphingomonas* and *Pseudomonas* (Figure 3). The pattern was quite different from a grass silage study (Franco et al., 2022), where the only correlation, and in that case positive, with the butyric acid concentration in the silages was the *Weissella* community, but this was not even identified in the current study.

*Fructilactobacillus* was negatively correlated only with pH (Figure 3) and, on the other hand, positively correlated with ammonia-N, lactic and acetic acids, total volatile fatty acids, total fermentation acids, total fermentation products and aerobic stability. Among these factors, the desirable correlations were mainly noticeable in the parameters of lower pH, higher lactic acid production and extended aerobic stability. It is worth mentioning that *Fructilactobacillus* was previously classified as *Lactobacillus* and for these and other reasons, species of the *Lactobacillus* genus are commonly used in ensiling experiments as silage additives (Merry et al., 2000; Filya, 2003; Eikmeyer et al., 2013; Oliveira et al., 2017; Guo et al., 2018; Keshri et al., 2018; Bai et al., 2020; Franco et al., 2022). Xu et al. (2021b) also indicated that, as expected, *Lactobacillus* (reclassified into many other genera including *Fructilactobacillus*) showed a negative correlation with pH and concomitant positive correlation with lactic and acetic acids, when studying ryegrass silages. Franco et al. (2022) identified a strong and positive correlation of *Lactobacillus* with acetic acid and aerobic stability, and speculated that there is a predisposition of this genus to improve the aerobic stability of silages by increasing the production of acetic acid in a controlled manner.

## Conclusion

Our results showed that crimped barley grains could be successfully ensiled under various MC and additive treatments. Low MC feeds have higher risk to be aerobically unstable while high MC results in more extensive fermentation, with potentially poor fermentation quality. The suitable additive depends on the raw material characteristics as LAB and SALT require relatively high MC to be effective, while FPA showed consistent improvements over all MC levels used in the current study. The MC assessment helps to identify the risks to the fermentative quality of the crimped ensiled grain, which allows for early preparation and use of an effective additive according to the prevailing conditions.

The bacterial growth in low MC was limited by low water activity, and thus only minor shifts in bacterial ecology were observed at low MC when compared to raw material with *Erwiniaceae* sp., Enterobacterales spp. and *Pseudomonas* dominating in both cases. However, a major change occurred from raw material towards medium and high MC silages, where *Fructilactobacillus* dominated the fermentation in CONT, FPA and SALT silages. LAB-treated silages at medium and high MC resulted in a distinguished pattern with dominance of *Lentilactobacillus* followed by *Lactiplantibacillus*. Both negative and positive correlations were identified between the bacterial communities and the fermentative characteristics of the silage.

Proper knowledge of bacterial populations and their interactions with silage characteristics is of great importance in designing the ensiling process and choosing the best management conditions to improve the efficiency of the production system and feed utilization. Additionally, the use of state-of-the-art technology such as next-generation sequencing to investigate the bacterial ecology of raw materials and silages can improve the development of additives for each environmental and management condition. These additives in turn can act synergistically with defined bacterial populations to improve the quality of silages in general.

## Data availability statement

The datasets presented in this study can be found in online repositories. The name of the repository and accession number can be found at: NCBI; PRJNA893459.

## Author contributions

MF contributed to conceptualization, methodology, formal analysis, visualization, data curation, and writing original draft, review and editing. IT contributed to methodology, formal analysis, visualization, data curation, and review and editing manuscript. MR contributed to conceptualization, methodology, review and editing, and funding acquisition. All authors have read and agreed to the published version of the manuscript.

## Funding

This work was supported by MiMi project funded by the Academy of Finland (grant number 322827).

## Conflict of interest

The authors declare that the research was conducted in the absence of any commercial or financial relationships that could be construed as a potential conflict of interest.

## Publisher’s note

All claims expressed in this article are solely those of the authors and do not necessarily represent those of their affiliated organizations, or those of the publisher, the editors and the reviewers. Any product that may be evaluated in this article, or claim that may be made by its manufacturer, is not guaranteed or endorsed by the publisher.
